# Emerging understandings of the role of exosomes in atherosclerosis

**DOI:** 10.1002/jcp.31454

**Published:** 2024-10-06

**Authors:** Zena Wehbe, Maya Wehbe, Ali Al Khatib, Ali H. Dakroub, Gianfranco Pintus, Firas Kobeissy, Ali H. Eid

**Affiliations:** ^1^ Vascular Biology Research Centre, Molecular and Clinical Research Institute St. George's University of London London United Kingdom; ^2^ Oxford University Hospitals Oxford United Kingdom; ^3^ Department of Nutrition and Food Sciences Lebanese International University Beirut Lebanon; ^4^ Departments of Medicine (Cardiology) and Population Health Science and Policy, Blavatnik Family Research Institute Icahn School of Medicine at Mount Sinai New York NY USA; ^5^ Department of Biomedical Sciences University of Sassari, Viale San Pietro Sassari 07100 Italy; ^6^ Department of Neurobiology, Morehouse School of Medicine Center for Neurotrauma, Multiomics & Biomarkers (CNMB) Atlanta GA USA; ^7^ Department of Basic Medical Sciences, College of Medicine QU Health, Qatar University Doha P.O. Box 2713 Qatar

**Keywords:** atherogenesis, cardiovascular disease, drug discovery, extracellular vesicles, phenotypic switch, vascular smooth muscle cells

## Abstract

Atherosclerosis remains a major contributor to cardiovascular disease, the leading cause of global morbidity and mortality. Despite the elucidation of several molecular, biochemical, and cellular aspects that contribute to the etio‐pathogenesis of atherosclerosis, much remains to be understood about the onset and progression of this disease. Emerging evidence supports a role for exosomes in the cellular basis of atherosclerosis. Indeed, exosomes of activated monocytes seem to accentuate a positive feedback loop that promotes recruitment of pro‐inflammatory leukocytes. Moreover, in addition to their role in promoting proliferation and invasion of vascular smooth muscle cells, exosomes can also induce neovascularization within lesions and increase endothelial permeability, two important features of fibrous plaques. Depending on their sources and cargo, exosomes can also induce clot formation and contribute to other hallmarks of atherosclerosis. Taken together, it is becoming increasingly evident that a better understanding of exosome biology is integral to elucidating the pathogenesis of atherosclerosis, and may thus provide insight into a potentially new therapeutic target for this disease.

## 1 INTRODUCTION

**Figure 1:**
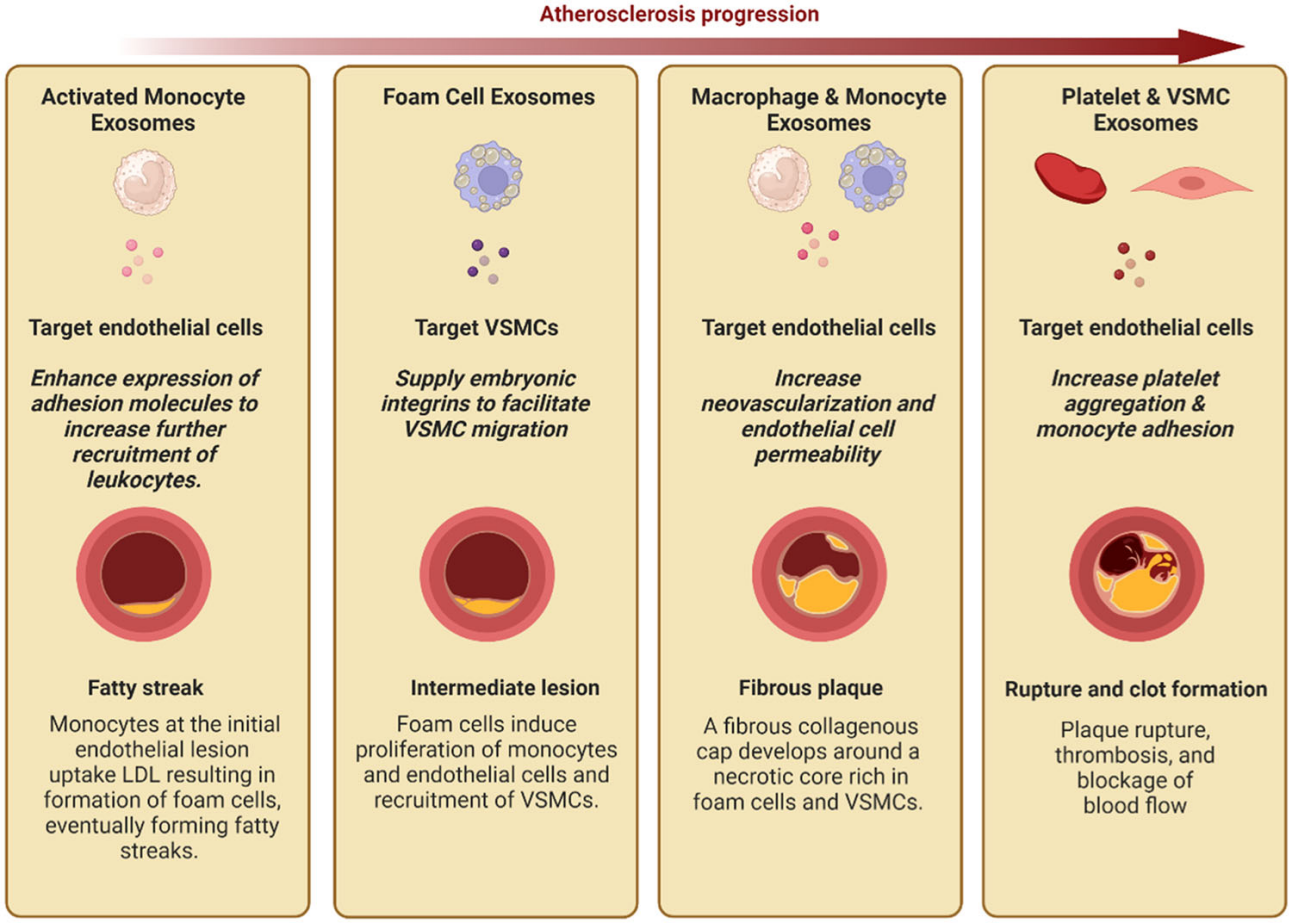
Throughout the various stages of atherosclerosis, exosomes from various cells facilitate the progression of the disease. Upon early fatty streak formation, exosomes from activated monocytes provide endothelial cells with adhesion molecules which enable recruitment of more monocytes and leukocytes to the site. As the fatty streak develops into an intermediate lesion, fat laden macrophages (foam cells) release exosomes which can supply embryonic type integrins to vascular smooth muscle cells (VSMCs) to allow their migration to the injured site. Further progression into a fibrous plaque coincides with an increase in exosome release from monocytes and macrophages which particularly target endothelial cells. This results in angiogenesis and further permeability of endothelial cells, eventually leading to the exposure of the collagenous cap into the arterial lumen. Subsequent platelet aggregation and clot formation is supported by exosomes secreted from platelets and VSCMs which target endothelial cells. Noting the important role of exosomes in the progression of atherosclerosis may provide further insight into specific characterization of the atherosclerotic stage, key molecular players involved in the pathophysiology of the disease, and more targeted therapeutic processes.

Atherosclerosis is the leading cause of coronary artery disease, strokes and periphery artery disease (Bentzon et al., 2014). Collectively, they are a result of a disruption in the integrity of the arterial lining. In addition to functioning as a semi‐permeable membrane for the exchange of nutrients and gases to the underlying arterial wall, the endothelium critically functions as a nonadhesive barrier. This feature lends itself to the normal unperturbed blood flow throughout the vasculature (Ross, 1995). However, this smooth passage is affected by arterial damage, such as that which is commonly caused by lipoprotein‐driven atherosclerosis (Figure 1) (Libby & Theroux, 2005). Specifically, oxidized LDL deposits in the sub‐endothelial layers develop into a fatty and fibrous plaque. Its eventual rupture through the wall results in a subsequent ‘sticky’ clot that narrows and hardens the lining. If untreated, this lesion may eventually culminate in the aforementioned cardiovascular diseases (Furie & Furie, 2008).

Atherosclerosis is initiated by the transit of lipoproteins, namely LDL, through the endothelium and into its underlying layers (Insull, 2009). The progress from these initial fatty streaks into fatal clots may span several decades via an inflammatory process which is uniform across all ages, races, and genders (Insull, 2009). However, the rate may be enhanced in genetically susceptible individuals and also depending on other confounding factors like smoking and obesity (Hong, 2010; Insull, 2009).

Dynamic micro‐communication between cells of the arterial wall and their environment ‐namely the underlying tunica and blood cargo‐ is essential for the manifestation of the disease (Saleh Al‐Shehabi et al., 2016). Major blood deliverables include LDL cholesterol, other lipids, and pro‐inflammatory cytokines, which are involved in each stage of disease development. However, it has become insufficient to examine cellular communication, physiological homeostasis, and disease development without considering the smallest extracellular vesicles known as exosomes. The purpose of this article is to elucidates the role of exosomes in the pathogenesis of atherosclerosis.

## 2 EXOSOMES

**Figure 2:**
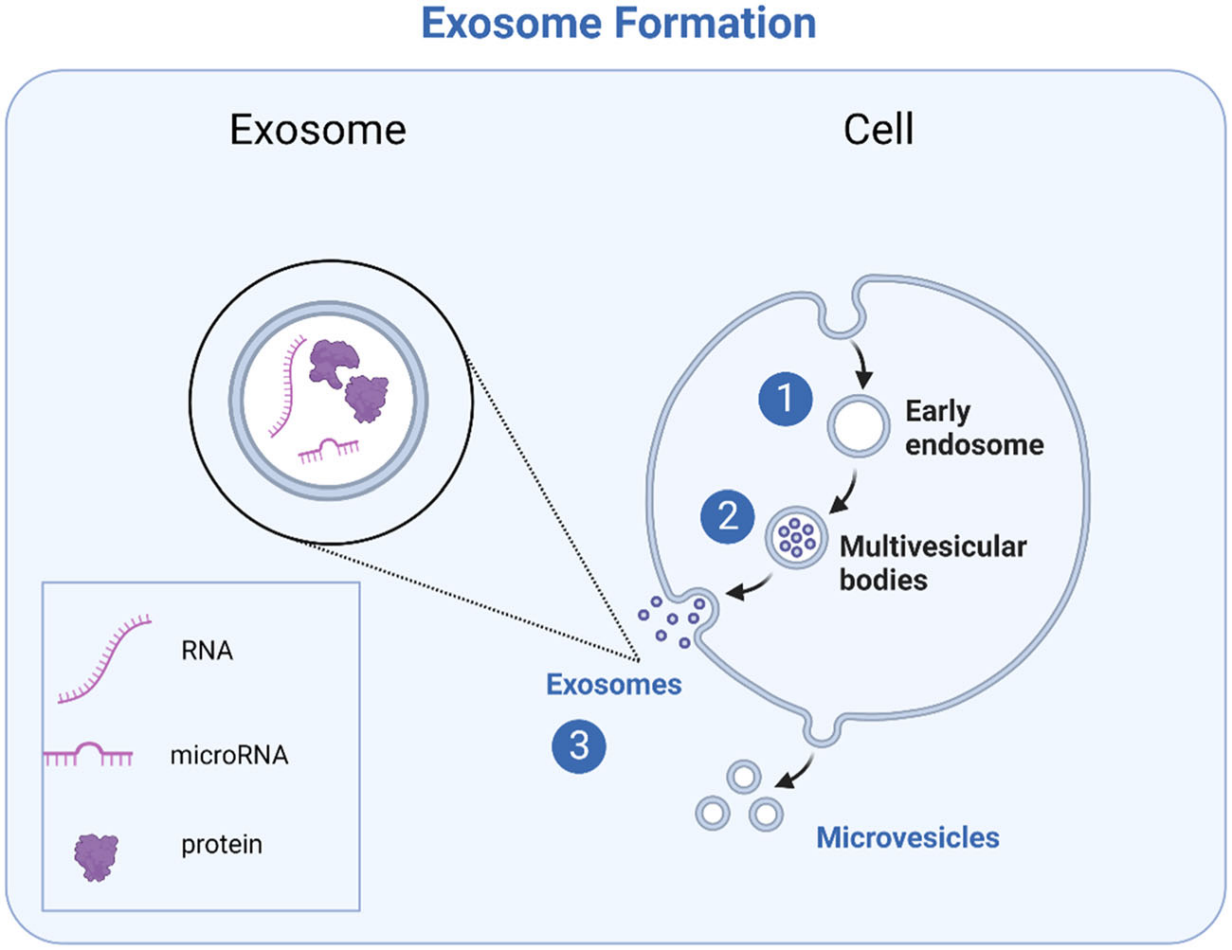
Exosomes formation. Exosomes are the smallest extracellular vesicles released by cells. Compared to other vesicles, exosomes are nanoparticles which are uniquely endosomal in origin. Their formation begins by the inward budding of the cell membrane, forming an endosome. The membrane of the endosome itself produces further inward invaginations, forming a multivesicular body (MVB) containing intraluminal vesicles. The MVB may fuse with lysosomes for degradation or it may be delivered to the cell membrane. Fusion of the MVB with the membrane causes released of the enclosed exosomes. In contrast, another type of extracellular vesicle, the larger microvesicle, is released by outward budding of the cell membrane.

The smallest size of extracellular vesicles secreted by cells are exosomes, and they are increasingly recognized as mediators for cellular communication (Marbán, 2018). Initially thought of as mechanisms to discard cellular waste, exosomes are increasingly recognized as vehicles useful for proximal and distant cellular communication (Abels & Breakefield, 2016). These vesicles are approximately 60 nm in size and have a saucer‐like morphology. Unlike microvesicles which are larger and form by outward budding the cell membrane, exosomes are uniquely endosomal in origin (Abels & Breakefield, 2016). Their formation begins as the cell membrane or endoplasmic reticulum pinch off into cytoplasm as endosomes. These will further invaginate, eventually creating a multivesicular body enclosing several intraluminal vesicles (ILVs). ILVs may be destined for degradation by lysosomes or fuse with the cell membrane and enter circulation (Anand et al., 2019). Although the sorting mechanism of ILVs is not completely elucidated, it is clear that an exosomal fate involves re‐organizing of its membrane to cluster together tetraspannins like CD63 and CD9 (Abels & Breakefield, 2016). During exosome formation, cytosolic contents, excluding organelles become enclosed within **(**Figure 2 **)** (Théry et al., 2002).

Exosomes effectively distribute an abundance of various cargo, protecting them from heat, RNase activity, and acidity due to their robust bilayer. In general, the cargo of exosomes differs according to species, cell source, and whether or not the cell is healthy or diseased (Anand et al., 2019). However, they do share common features especially those involved in their formation, secretion, and uptake (Simons & Raposo, 2009). Their contents broadly include proteins, lipids, and nucleic acids namely microRNA (Anand et al., 2019). Proteins include tetraspanins, integrins, transmembrane proteins, cytosolic proteins like actin, and more. Importantly, Fitzgerald et al., identified that exosomes also peripherally display and enclose a broad array of cytokines (Fitzgerald et al., 2018). In fact, depending on the cytokine and cell type, exosomes will preferentially transport them as membrane‐bound or intracellular soluble forms. Cholesterol, sphingomyelin, and ceramides constitute a major portion of exosomal lipids mostly localized to lipid rafts on the exosome membrane (Théry et al., 2002). An abundance of microRNAs (miRs) is also enclosed within the vesicles.

Over 60% of genes are regulated by microRNA (miR) that commonly modulate mRNAs involved in growth, differentiation, and the immune response (Chen et al., 2012). They function primarily by binding to mRNA and preventing its translation. MiRs are especially important in the proliferation and maturation of lymphocytes and monocytes. Lack of certain miRs has been implicated in immunodeficiency, arthritis, and impaired perinatal cardiac development (Pauley et al., 2009). Importantly, the miR library of exosomes is altered when the cell source is diseased (Jia et al., 2014).

Once exosomes reach the target cells, they are either internalized by endocytosis, fuse with the membrane, and release their contents into the cytosol or exert an effect via ligand‐ligand interactions (Abels & Breakefield, 2016; Anand et al., 2019). The following summarizes how these unique features of exosomes have shaped them as key factors in atherosclerosis progression and characterization.

## 3 EXOSOMES IN THE VARIOUS STAGES OF ATHEROSCLEROSIS

### 3.1 Fatty streaks

In a healthy individual, the arterial lumen diameter is maintained by a balance of vasoconstrictive and vasodilatory factors. For example, nitric oxide and prostaglandins promote vasodilation and anti‐coagulation, while simultaneously present endothelin is vasoconstrictive (Carew et al., 1987; Dzau, 1990). Moreover, endothelial production of heparin sulfate and cell surface ectoADPase also confer protection against coagulation (Ross, 1995). Factors that perturb the regulation of lumen diameter, coagulation, and shear force, like hypertension and hyperlipidemia, can contribute to the increase in permeability of the endothelial cells (ECs) (Ross, 1995). As lipoproteins increasingly transit through the permeabilized endothelium, they are oxidized by lipoxygenases that render them pro‐inflammatory (Insull, 2009). The oxidized LDL upregulates the expression of endothelial wall adhesion molecules like intercellular adhesion molecule‐I (ICAM‐I) (Kevil et al., 2001). These display a significant affinity for blood leukocytes, primarily monocytes and to a lesser degree T lymphocytes, and facilitate their subsequent passage into the intimal connective tissue (Insull, 2009; Tabas, 1994). Here monocytes become activated macrophages (MACs) and, along with T lymphocytes, release inflammatory cytokines like prostaglandins and histamine (Libby & Theroux, 2005; Yang et al., 2014). They play a major role in the inflammatory response due to the copious amounts of cytokines and growth factors they release (Hong, 2010). Importantly, MACs transform into lipid‐laden foam cells after uptake of the intimal LDL, eventually forming microscopically detected yellow fatty streaks (Figure 1) (Ross, 1995; Stary, 1990). Even during these early stages of atherosclerosis, exosomes have been shown to play a role.

The main source of exosomes involved in fatty streak formation are those released by monocytes. Tang et. al demonstrated that the content of monocyte‐derived exosomes is modified upon pro‐inflammatory stimulation by interferon‐gamma (INF‐ɣ) and lipopolysaccharide (LPS) (Tang et al., 2016). Particularly, microRNAs involved in the inflammatory response of monocytes, miR‐155 and miR‐233, are significantly altered within these exosomes. The target of these exosomes is primarily endothelial cells. Human umbilical vascular ECs(HUVECs) have been shown to effectively internalize these bioactive vesicles that subsequently cause an increase in the expression of Nuclear Factor‐kappa B (NF‐κB) (Tang et al., 2016). NF‐κB is a transcription factor closely involved with cytokine production and cell survival (Gilmore, 2006). Not only is it elevated in the HUVECs, but its target genes are also modified. Specifically, Intercellular Adhesion Molecule 1 (ICAM‐1) and Chemokine (C‐C motif) Ligand 2 (CCL2) are significantly expressed. Monocytes rely on ICAM‐1 to adhere to ECs (Kevil et al., 2001). As previously discussed, expression of adhesion molecules by ECs is necessary to draw in monocytes and T lymphocytes in towards the intima, eventually producing a fatty streak. CCL‐2 is usually secreted by monocytes to attract further monocytes and T lymphocytes to a site of infection or injury (Carr et al., 1994). It is upregulated in vascular ECs in the presence of pro‐inflammatory stimuli and is highly correlated with atherosclerotic plaque formation (Khyzha et al., 2019). As such, exosomes of activated monocytes provide an additional dimension to positive feedback recruitment of these leukocytes, primarily through their action on endothelial cells.

### 3.2 Intermediate lesions

Once fatty streaks have formed, they may regress or transition into intermediate lesions, which are mostly localized at the vulnerable branch points of the vasculature (Tabas, 1994). As MACs uptake lipids, production of cytokines and growth modulators increases, leading to the proliferation of monocytes and ECs as well as the recruitment of VSMCs from the tunica media (Insull, 2009). Most of the arterial wall is composed of VSMCs that are both in contact with the thinner intimal and thicker adventitial connective tissue and thus direct most of the tonality (Ross, 1995). In addition to pro‐inflammatory cytokines, chemotactic factors released by ECs also recruit VSMCs into the intima (Libby et al., 1988). Importantly, integrins are involved in the migration of VSMCs. Atherogenic VSMCs in adults revert to the expression of embryonic integrins, like α2β1, on their surface rather than the normal adult α1β1. In contrast to α1β1 VSMCs, those with embryonic integrins demonstrate the ability to migrate (Skinner et al., 1994). VSMCs proliferate and release matrix metalloproteinases (MMPs) that weaken the intima's connective tissue, namely collagen fibrils, and elastin, while also depositing new ECM proteins such as proteoglycans (Bentzon et al., 2014). Proteoglycans increasingly hone in more LDL which is taken up by MACs as well as VSMCs (Insull, 2009). Collectively, protein deposits by VSMCs, degradation of connective tissue, and an increase in LDL shape the fibro‐fatty lesion.

In the intermediate lesion, the predominantly featured exosomes are those derived from foam cells that target VSMCs. Compared to healthy individuals, those with atherosclerosis have a different pool of circulating exosomes which significantly increases the migration of VSMCs into the intima (Niu et al., 2016). This migration had been previously attributed to inflammatory‐induced production of soluble cytokines, growth factors, and chemotactic modulators released by the ECs and foam cells in a primarily paracrine manner. However, it has become clear that deliberately repackaged exosomes also play a role.

Interestingly, foam cells (FCEx) release almost 2.5 fold more exosomes relative to exosomes of MACs and display a more pronounced effect on VSMC migration and adhesion (Niu et al., 2016). Proteomic analysis indicated that foam cell exosomes carried almost double the amount of proteins as those in macrophage‐derived exosomes (Mex). The majority of the FCex proteins are involved in the actin cytoskeleton and focal adhesion and exert their effect via an ERK/AKT pathway (Niu et al., 2016).

Foam cell‐derived exosomes notably display integrins β1 and α5 and directly supply them to the VSMCs, without increasing endogenous mRNA production (Niu et al., 2016). Previously, it was discussed that VSMCs uniquely revert to embryonic integrins which facilitate their migration. This “reversion” to altered integrins may be attributed to the integrins delivered by the exosomes by way of fusion with the VSMC membrane.

In addition to the membrane‐bound integrins, miRs enclosed within the foam cell‐derived exosomes can also modulate VSMC activity (Ren et al., 2022). In line with this, exosomes from oxidized‐LDL stimulated MACs evoked a significant increase in exosome‐derived miR‐185‐5p in VSMCs (Ren et al., 2022). It was shown that this miR can inhibit the PI3K/AKT/mTOR pathway, leading to enhanced VSMC proliferation and invasion, thus potentially accelerating atherosclerosis (Ren et al., 2022). Moreover, recent evidence shows that macrophage‐derived miRs can modulate endothelial cell behavior and promote endothelial injury(Liu et al., 2022). These findings highlight the important role of foam cell‐derived exosomes in shaping VSMC contribution to fibrous plaque development.

### 3.3 Fibrous plaque formation

Ensuing modification of the lipoproteins, like glycation and oxidation, continues to propel the inflammatory response and lead to necrosis. A fibrous collagenous cap develops around a necrotic core rich in foam cells and VSMCs (Libby & Theroux, 2005). Ultimately, the cells of the lesions are increasingly deprived of oxygen and nutrients, triggering angiogenesis from existing vasa vasorum (Krock et al., 2011). Hypoxia‐inducible transcription factors (HIF) in ECs upregulate proteins involved in various pathways of angiogenesis (Krock et al., 2011). In particular, vascular endothelial growth factor A (VEGF‐A) increases the permeability and migration of the ECs and causes them to protrude filopodia appendages which guide them to the target location where angiogenesis is required (Krock et al., 2011; Potente et al., 2011). Production of these micro‐vessels within the lesion enable the delivery of oxygen, nutrients, MACs, lipids, and inflammatory modulators (Camaré et al., 2017).

In a process that resembles embryonic osteogenesis, bone‐like calcifications can also form within a plaque (Abedin et al., 2004). Many osteogenic proteins such as osteopontin and bone morphogenic protein 2 (BMP‐2) are detected within lesions. MACs can differentiate into osteoclast‐like cells upon encounter with calcium deposits and pro‐inflammatory cytokines (Abedin et al., 2004; Merkel et al., 1999). Mineralization is also possible due to the increase in oxidized LDL within the intima. An increase in calcium deposits is associated with further hardening and dysfunction of the arterial wall (Abedin et al., 2004). Collectively, these events, including neovascularization and proteolytic degradation of the fibrous cap, weaken the intima, eventually causing the rupture and protrusion of the lesion into the arterial lumen (Camaré et al., 2017; Furie & Furie, 2008; Ross, 1995; Weaver, 2013).

Within the actual plaques, the largest population of exosomes are those derived from MACs and monocytes (Figure 1). The exosomes from MACs and monocytes mainly deliver miRs to recipient cells like other monocytes and ECs(Hulsmans & Holvoet, 2013). In vitro and in vivo experiments confirmed that human ECscan rapidly uptake monocyte‐derived exosomes (Zhang et al., 2010). Moreover, some miRs, like miR‐150, can also be retained and detected within the target cells in significantly high levels (~12‐fold higher), suggesting intact delivery of miRs (Zhang et al., 2010). This miR‐150 is known to be active within the endothelial cell as it inhibits the expression of transcription factor c‐Myb (Zhang et al., 2010). This results in increased endothelial migration and increased vascularization of the fibrotic plaque. Incidentally, atherosclerosis patients have elevated levels of enclosed circulating miR‐150 (Qiu et al., 2020). It is important to mention here that recent evidence shows that miR‐150 delivery to ECs could be protective, evidenced by the ability of this miR to suppress the expression of pro‐inflammatory, proapoptotic, or pro‐fibrotic genes (Russomanno et al., 2021). Early evidence suggested that miR‐150 induces apoptosis in ECs (Qin et al., 2017). These contradictory effects may be attributed to the model employed or the vascular bed from where these ECs were isolated. This warrants that extreme care must be taken when analyzing results from these studies, and that endothelial cell models be appropriately selected for the disease model investigated. Monocyte‐derived exosomes induce neovascularization within lesions and increase endothelial permeability promoting fibrous plaque formation. Eventually, the exposed collagenous cap protruding from the lesion triggers a surge of platelets to the site of injury.

### 3.4 Clot formation

Upon exposure of the exposed collagenous cap, the bound von Willebrand factor adheres to glycoprotein VI on the surface of circulating thrombocytes, triggering a surge of platelets to the site of injury (Furie & Furie, 2008). Tissue factor (TF) is a membrane‐bound protein on cells of the adventitia and on VSMCs of the tunica media which can adhere to the platelets. By also binding to the serine protease Factor VII, TF cleaves pro‐thrombin, generating thrombin and ultimately fibrin (Furie & Furie, 2008). Subsequently, thrombin activates platelets by cleaving their membrane‐bound protease‐activated receptor 1 (Par1), enabling the release of molecules such as thromboxane‐A2 and adenosine monophosphate, causing a positive feedback mechanism that draws more platelets to the site (Furie & Furie, 2008). If left untreated, the expanding clot manifests clinically as stroke, myocardial infarction, peripheral arterial disease, and more (Libby & Theroux, 2005).

Factors like cytokines, growth regulators, and mechano‐transduction link the events occurring in the lumen to phenotypic changes at every level of the arterial wall. The ability of the microenvironment to orchestrate a stepwise progression from fatty streak to obstructive thrombus stems from communication between all the elements of the arterial layers and lumen.

Contrary to the previous steps, a variety of exosomes from platelets and VSMCs play major roles in thrombus formation. Platelet‐derived extracellular vesicles, including exosomes (PEx), are especially elevated in the circulation of advanced atherosclerosis patients (Suades et al., 2012). They target mainly endothelial and intimal cells at the site of the thrombus. Though the mechanism is not established, PEx significantly increased platelet coagulation and fibrin deposition on damaged arterial walls in in vitro conditions (Suades et al., 2012). Moreover, they have also been shown to specifically increase the production of ICAM‐1 by ECs resulting in a direct increase in monocyte adhesion (Barry et al., 1998).

In addition to endothelial cells, PEx can target platelets and VSMCs as well. The surface protein P‐selectin on the surface of PEx interacts with its corresponding P‐selectin receptor on platelets and upregulates the production of TF (Furie & Furie, 2004). Thrombin‐induced platelets release exosomes containing copious amounts of miR‐223, miR‐21, and miR‐339 (Tan et al., 2016). Once internalized by VSMCs, they repress the production of platelet‐derived growth factor beta (PDGF‐β) leading to apoptosis and further instability of the plaque (Tan et al., 2016)

Of all the exosomes released, those secreted by VSMCs display the most potent thrombogenic effect probably due to the relative abundance of both TF and phosphatidyl serine (PS) on their membrane (Kapustin et al., 2017). Due to its negative charge, PS can adhere to thrombogenic factors like circulating prothrombin (PT) and Factors VII and X among others. This places them in close proximity to TF, facilitating their cleaving and thereby activating the coagulation signaling cascade (Kapustin et al., 2017).

## 4 EXOSOMES AND ATHEROSCLEROSIS PATHOGENESIS

Exosomes modulate the onset and progression of atherosclerosis through modulating intercellular signaling. The interaction between MACs and ECs following endothelial injury plays a major role in the onset and progression of atherosclerosis. MACs are among the first cells to migrate to the injured endothelium and release various inflammatory molecules via exosomes that impact EC functions. These exosomes contain cytokines and chemokines like tumor necrosis factor‐alpha (TNF‐α) and interleukin‐6 (IL‐6) (Yu & Wang, 2019). TNF‐α activates ECs to facilitate leukocyte adhesion and transmigration into the vessel wall by enhancing the expression of adhesion molecules like ICAM‐1 (Bui et al., 2020). IL‐6 enhances the inflammatory response by stimulating the production of acute‐phase reactants from the liver (Choy & Rose‐John, 2017). In addition to cytokines and chemokines, exosomes contain microRNAs (miRNAs) or exosomal microRNAs (exomiRs), both of which can impart a proatherogenic or antiatherogenic response depending on the exosomal cargo and cells involved (Table 1).

**Table 1:** The role of exosomes in atherosclerosis.

Ref.	Cargo	Intercellular communication (Donor cell – recipient cell)	Target/Signaling Pathway	Function
*Proatherogenic*				
Chang et al. (2019); Loyer et al. (2014)	miR‐92a	ECs – MACs	KLF4	Promotes formation of atherosclerotic plaque
Li et al. (2020)	CD137	ECs – VSMC	TET2	Promotes VSMCs proliferation and migration and neointimal formation
Kapustin et al. (2015)	Noncrystalline Ca/P salt	ECs – VSMC	SMPD3	Promotes VSMCs calcification
Sun et al. (2012)	miR‐155	HUVECs – HUVECs	eNOS	Promotes diastolic dysfunction of blood vessels
Niu et al. (2016)	integrins	MACs – VSMCs	ERK/AKT	Promotes VSMC migration and adhesion by altering phosphorylation levels
Zhu et al. (2019)	miR‐21‐3p	MACs – VSMCs	PTEN	Promotes VSMC migration and proliferation
Liu et al. (2020)	miR‐106a‐3p	MACs – VSMCs	NA	Promotes VSMCs proliferation and inhibits VSMCs apoptosis
Nguyen et al. (2018)	miR‐146a	Atherogenic MACs – naïve MACs (paracrine signaling)	IGF2BP1/HuR	Accelerates progression of atherosclerosis by reducing MAC migration
Sun et al. (2019)	miR‐126	Platelets – HUVECs	NA	Promotes intraplaque angiogenesis by stimulating ECs proliferation and migration
*Antiatherogenic*				
He et al. (2018)	miR‐155	ECs – MACs	KLF2/miR‐155	Inhibits inflammatory reaction
Hergenreider et al. (2012)	miR‐143/145	ECs – VSMC	KLF2	Reduces atherosclerotic lesion formation by controlling VSMC phenotypes
Njock et al. (2015)	miR‐10a	ECs – monocyte	NF‐κB	Represses inflammation by regulating monocyte activation
Heo et al. (2020)	miR‐1246miR‐182miR‐486	VSMC – ECs	NA	Maintains vascular homeostasis by inhibiting EC migration
Bouchareychas et al. (2020)	miR‐99amiR‐146b miR‐378a	Naive bone marrow‐derived MACs– MACs	NF‐κB/TNF‐α	Reduces necrotic lesion area, and stabilizes atheroma by promoting M2 polarization, and reducing hematopoiesis
Zhang et al. (2019)	miR‐146a	MACs – Neutrophils	SOD2	Slows atherosclerosis progression by promoting ROS and NETs release
Li et al. (2019)	miR‐let7	MSCs – MACs	IGF2BP1/PTEN HMGA2/NF‐κB	Promotes M2 polarization
Xing et al. (2020)	miR‐342‐5p	MSCs – ECs	PPP1R12B	Protects against atherosclerosis
Li et al. (2017)	miR‐223	Platelets –HUVECs	NF‐κB/MAPK	Regulates thrombosis‐inflammation reaction by inhibiting ICAM‐1 expression
Zhong et al. (2019)	miR‐146a	DCs – ECs	IRAK‐1	Regulates inflammation response

Abbreviations: AKT, protein kinase B; DC, dendritic cells; ECs, endothelial cells; eNOS, endothelial nitric oxide synthase; ERK, extracellular regulated protein kinases; HMGA2, high mobility group A; HuR, human antigen R; HUVECs, human umbilical vein endothelial cells; ICAM‐1, intercellular adhesion molecule‐1; IGF2BP1, insulin‐like growth factor 2 mRNA‐binding protein 1; IRAK‐1, interleukin‐1 receptor‐associated kinase‐1; KLF2, krüppel‐like factor 2; KLF4, krüppel‐like factor 4; M2, M2 macrophages; MACs, macrophages; MAPK, mitogen‐activated protein kinase; MSCs, mesenchymal stem cells; NA, not available; NETs, neutrophil extracellular traps; NF‐κB, nuclear factor‐κB; PPP1R12B, protein phosphatase 1 regulatory subunit 12B; PTEN, phosphatase and tension homolog; ROS, reactive oxygen species; SMPD3, sphingomyelin phosphodiesterase 3; SOD2, superoxide dismutase 2; TET2, ten‐eleven translocation 2; TNF‐α, tumor necrosis factor α; VSMC, vascular smooth muscle cell.

### 4.1 Proatherogenic effects of exosomes

One of the initial stages of atherosclerosis is endothelial injury. Following this insult, ECs increase the release of exomiR‐92a (Loyer et al., 2014). This exomiR is potent enough to drive the formation of atherosclerotic plaque by virtue of its ability to activate MACs through a targeted regulation of krüppel‐like factor 4 (KLF4) (Chang et al., 2019; Loyer et al., 2014). Interestingly, it is through exosomes that a consortium of cells, namely ECs, MACs, and VSMCs, interact with each other during the formation of atherosclerotic plaque. For instance, EC‐derived exosomes can promote neointimal formation and VSMC proliferation and migration by altering VSMC phenotype switching through a CD137‐ dependent signaling pathway (Li et al., 2020). In addition, VSMC‐derived exosomes can impact vascular hemostasis by promoting VSMC calcification (Kapustin et al., 2015). Moreover, macrophage foam cell‐derived exosomes have been shown to enter VSMCs and transfer integrins to promote VSMC migration and adhesion through an ERK/AKT‐dependent pathway (Niu et al., 2016). In this context, some molecules, like nicotine, can promote the proliferation and migration of neighboring VSMCs by inducing MACs to release exomiR‐21‐3p via a PTEN‐dependent mechanism (Zhu et al., 2019).

Exosomes may also play an important role in vascular endothelial function. For instance, miR‐155 is well known to be upregulated in oxidized‐LDL‐treated macrophages and atherosclerosis plaques (Nazari‐Jahantigh et al., 2012; Urbich et al., 2008). miR‐155 has been shown to promote endothelial dysfunction by inhibiting endothelial nitric oxide synthase (eNOS) expression and decreasing endothelium‐dependent vasorelaxation (Sun et al., 2012). Moreover, oxidized LDL‐stimulated macrophage has been shown to release miR‐106a‐3p which inhibit VSMC apoptosis and promotes VSMC proliferation (Liu et al., 2020). In addition, activated platelet‐derived exomiR‐126 was shown to enhance intraplaque angiogenesis by stimulating the proliferation and migration of HUVECs (Sun et al., 2019).

### 4.2 Antiatherogenic effects of exosomes

Exosome‐mediated interactions can exert an antiatherogenic response (Table 1). For instance, oxidized LDL or overexpression of KLF2 resulted in enhanced exomiR‐155 levels in ECs (He et al., 2018). ExomiR‐155 represses the inflammatory response by polarizing MACs to M2 cells (He et al., 2018). Moreover, exomiR‐143/145 derived from KLF2‐expressing ECs have been shown to reduce atherosclerotic lesions in ApoE‐/‐ mice by regulating VSMC phenotype (Hergenreider et al., 2012). Another mechanism by which EC‐derived exosomes attenuate the inflammatory response involves the transfer of exomiR‐10a to regulate monocyte activation by targeting an NF‐κB‐dependent signaling pathway (Njock et al., 2015).

Similarly, VSMCs can protect against atherosclerosis through its exosomal‐mediated interaction with ECs. VSMCs lie below the endothelium, and at physiological conditions regulate vascular tension (Basatemur et al., 2019). VSMC‐derived exosomes are thought to influence vascular hemostasis (Kapustin et al., 2015; Qiu et al., 2018). For instance, exomiR‐1246, exomiR‐182, and exomiR‐486 secreted by VSMCs have been shown to regulate vascular homeostasis by inhibiting EC migration (Heo et al., 2020). MACs, on the other hand, reside in the subendothelial space of the arterial wall and are involved in all the stage of atherosclerosis including endothelial injury, endothelial dysfunction and plaque formation (Tabas & Bornfeldt, 2016). For example, exomiR‐99a, exomiR‐146b, and exomiR‐378a derived from activated MACs were found to reduce necrotic lesion area and stabilize atheroma by downregulating TNF‐α/NF‐κB signaling (Bouchareychas et al., 2020).

In addition, exosomes modulate the interaction between mesenchymal stem cells (MSCs), platelets, or dendritic cells (DCs) with ECs during atherosclerosis. For instance, adipose‐derived MSCs secrete exomiR‐342‐5p that exerts an anti‐atherosclerotic effect on ECs (Xing et al., 2020). Additionally, exomiR‐223, exomiR‐339, and exomiR‐21 released by platelets increased following thrombin activation (Li et al., 2017). Contextually, exomiR‐223 suppresses EC inflammation by inhibiting the expression of TNF‐α ‐stimulated ICAM‐1 (J. Li et al., 2017). Moreover, exomiR‐146a released by DCs can also modulate inflammation in ECs by inhibiting interleukin‐1 receptor‐associated kinase (IRAK)‐1 (Zhong et al., 2019).

### 4.3 Exosomes and therapeutic targets

Exosomes are emerging as potential biotherapeutics and drug delivery vectors as they can target several signaling pathways across all stages of atherosclerosis. As such, exosomes could be utilized to enhance the therapeutic delivery of RNAs, peptides, and synthetic drugs (Barile & Vassalli, 2017). For instance, one possible therapeutic approach for atherosclerosis involves using an exosome‐mediated *Ldlr* mRNA delivery strategy which has been established in a mouse model (Li et al., 2021). This method could effectively restore *Ldlr* expression and stabilize atherosclerotic plaques. In addition, exosomes may sever as potential biotherapeutics for cardiac diseases. For example, exosomes retrieved from cardiac stem cells could promote better regeneration in recipient cardiac cells (Zamani et al., 2019). Stem cell‐derived exosome administration in mouse models has been shown to be a promising strategy for the treatment of cardiovascular diseases (Suzuki et al., 2016). For instance, the treatment of ApoE^−/−^ mice with MSC‐derived exomiR‐let7 decreased the atherosclerotic plaque size, promoted M2 macrophage polarization through the HMGA2/NF‐κB pathway, and suppressed macrophage infiltration via the IGF2BP1/PTEN pathway (Li et al., 2019).

However, the utilization of stem cell‐derived exosomes remains limited due to lack of well‐defined manufacturing platforms (Colao et al., 2018). Moreover, some exosomes can exert dual atherogenic effects depending on their cellular origin and the surrounding environment. For example, exomiR‐146 from atherogenic macrophages slowed atherosclerosis progression by enhancing the release of reactive oxygen species and neutrophil extracellular trap (Zhang et al., 2019). In contrast, exomiR‐146a secreted from macrophages in a proatherogenic environment reduced MAC migration and possibly accelerated atherosclerosis progression (Nguyen et al., 2018). Consequently, the administration of stem cell‐derived exosomes and their therapeutic application may be challenging due to the variability in their effects, which depend on the pathological environment and severity of the disease.

Nonetheless, targeting miRNA content in exosomes may be more effective for treating atherosclerosis compared to targeting serum RNAs, as exomiRNAs are more stable. For example, endothelial‐specific miR‐126 has been shown to maintain vessel integrity and thus can be used to enhance vascular repair and regeneration (Wang et al., 2008). In addition, inhibition of miR‐155 could improve endothelial dysfunction by enhancing endothelium‐dependent vasorelaxation (Sun et al., 2012). Although not originally intended, targeting miRNAs may also contribute to the mechanisms of existing drugs. For example, Simvastatin has been shown to improve TNF‐α‐induced endothelial dysfunction by downregulating miR‐155 expression (Sun et al., 2012). Other exomiRNAs involved in atherosclerosis like exomiR‐92a, exomiR‐21‐3p, exomiR‐106a‐3p, and exomiR‐126 are potential targets to alleviate the atherosclerotic burden (Table 1). However, the exact impact of each proatherogenic exomiRNA on the total disease burden remains unknown and warrants further investigation. ExomiRNA‐21 is the most abundant miRNA in MACs and is known to enhance inflammatory cytokines, such as IL‐10 (Caescu et al., 2015; Das et al., 2014). Thus, modifying exomiR‐21 in MACs might be a promising therapeutic target to effectively reduce atherosclerotic burden.

Despite all challenges, exosomes are emerging as promising biotherapeutics. Table 1. summarizes some of the well‐established exomiRNAs, including their targets and functions. Further research is required to determine the most effective exomiRNA for therapeutic targeting.

## 5 CONCLUSIONS AND PERSPECTIVES

The seemingly stage‐specific exosomes that are active throughout the progression of atherosclerosis also means they may be used as biomarkers for the disease. In fact, many studies have demonstrated that miRs produced and packaged into exosomes are altered during an inflammatory response (Croce, 2009; Gillan et al., 2019; Zidar et al., 2016). This feature has enabled miR‐derived exosomes to serve as both diagnostic and prognostic tools (Wang et al., 2019). For example, inflammation induced by LPS or streptozotocin and mediated by toll‐like receptor 4 (TLR‐4) significantly increased the production of miR‐181a by MACs (Xie et al., 2013). During hypoxic stress, elevated mir‐181a directly targets and inhibits the mRNA of bcl‐2, a key apoptotic protein (Liu et al., 2016). Consequently, ECs that uptake miR‐181a and suppress bcl‐2 translation, have a reduced tolerance to hypoxia and undergo apoptosis. Incidentally miR‐181a is also visibly elevated in the circulation of atherosclerosis patients (Xie et al., 2013). Moreover it is remarkably overexpressed in the plaque itself as a result of hypoxia (Liu et al., 2016). As such miR‐181a is a proposed biomarker of atherosclerosis (Xie et al., 2013).

In addition to the localized modulations that exosomes exert during the various stages of atherosclerosis, extracellular vesicles (EVs), which include exosomes, may induce atherosclerotic signals at distant sites. Similar to the role of exosomes in cancer metastasis, EVs can deliver their cargo to distant tissues via circulation. It has been shown that EVs derived from hepatocytes promote vascular inflammation and atherogenesis through microRNAs (Jiang et al., 2020). More relevantly, EVs isolated from atherosclerotic rats could increase the in vitro expression of E‐selectin, VCAM‐1 and ICAM‐1 in ECs (Peng et al., 2022). These EVs can induce in vivo arterial wall thickening, intimal inflammation and narrowing of carotid lumens (Peng et al., 2022). Apparently, these atherosclerosis‐derived EVs carry a significantly higher level of miR‐23a‐3p than those derived from non‐atherosclerotic animals (Peng et al., 2022). This miR promotes endothelial inflammation and lumen narrowing, while its antagomir reverses the effect of the atherosclerotic EVs (Peng et al., 2022). This clearly highlights the role EVs may play in the ‘metastasis’ of atherosclerosis and that specific miRs may be directly responsible for this effect.

There are not enough substantial studies demonstrating how exosomes are used as therapy to manage atherosclerosis. Predominantly expressed or inhibited proteins‐as a result of exosome treatment‐ offer insight into proteins that can be targeted to manage the disease (Y. Wang et al., 2019).

For example, in both in vitro and in vivo models, miR‐126 was shown to reduce expression of pro‐inflammatory cytokines in MACs exposed to oxidized LDL (Hao & Fan, 2017). Furthermore, mice treated with miR‐126 showed a significant reduction macrophage accumulation at the plaque site and a decrease in the area of the lesion (Hao & Fan, 2017). Therefore miR‐126 found abundantly in exosomes appears to be a promising tool in decreasing the inflammatory response of a growing plaque (Hao & Fan, 2017).

There is little doubt that exosomes possess a functional role in atherosclerosis progression. As such, examining and comparing the contents of exosomes can offer insight into the key active players during each stage. Understanding the complete profile of atherosclerotic exosomes, their targets, and the ensuing phenotypic responses would provide a better understanding of their functional value, and offer relevant and specific therapeutic targets. Given the important role exosomes contribute to atherosclerosis progression, it still remains to be investigated if targeted suppression of exosomal release may decelerate this process. Because cells that secrete exosomes are also involved in other inflammatory diseases, it is only warranted to examine the role of these EVs in progression of various diseases.

## AUTHOR CONTRIBUTIONS

Zena Wehbe, Maya Wehbe, AA, Ali Dakroub, Gianfranco Pintus, Firas Kobeissy and Ali H. Eid contributed to the writing. Ali H. Eid contributed to the writing and edited the final version of the manuscript. Ali H. Eid was responsible for conceptualization as well as project administration, coordination and supervision.
